# Explaining sexual self-care status and its predictor factors in women referring to healthcare centers of Sari, Iran, 2021

**DOI:** 10.1186/s12905-023-02196-4

**Published:** 2023-02-08

**Authors:** Fereshteh Yazdani, Masoumeh Simbar, Zeinab Hamzehgardeshi, Elham Zare, Malihe Nasiri

**Affiliations:** 1 Student Research Committee, Department of Midwifery and Reproductive Health, School of Nursing and Midwifery, Shahid Beheshti University of Medical Sciences, Tehran, Iran; 2 Department of Midwifery and Reproductive Health, Midwifery and Reproductive Health Research Center, School of Nursing and Midwifery, Shahid Beheshti University of Medical Sciences, Tehran, Iran; 3 Sexual and Reproductive Health Research Center, Department of Reproductive Health and Midwifery, Faculty of Nursing and Midwifery, Mazandaran University of Medical Sciences, Sari, Iran; 4 Department of Basic Sciences, Faculty of Nursing and Midwifery, Shahid Beheshti University of Medical Sciences, Tehran, Iran

**Keywords:** Self-care, Sexual self-care, Women, Sexual health

## Abstract

**Introduction:**

With the increase in the prevalence of non-communicable diseases and chronic health problems along with population growth, one of the definitions that is expanding is the ability to adapt and self-manage against diseases and self-care. Although there are several studies to examine self-care in medical conditions, there are not enough concepts and data related to sexual self-care. Therefore, the present study was conducted with the aim of explaining the status of sexual self-care and its predictor factors in women of reproductive age referring to healthcare centers.

**Material and methods:**

This research was a cross-sectional study that was conducted on 400 women of reproductive age that referring to healthcare centers affiliated to Mazandaran University of Medical Sciences, Sari in 2021. Data were collected using personal-obstetric characteristics and sexual self-care questionnaires in women of reproductive age. Pearson correlation coefficients, independent t-test, one-way analysis of variance and multiple linear regression model were used to analyze the data.

**Results:**

The average total score of sexual self-care in reproductive age women participating in the research was 70.66% ± 12.52%. In addition, the prevention of women's cancers and the prevention of unintended pregnancies obtained the lowest and highest scores, respectively. Factors such as age, education, education related to medical sciences, history of HIV testing, history of HPV vaccination, source of sexual information, method of contraception and infection-related action in a person can affect the level of sexual self-care in women. Based on the final regression model, education related to medical sciences (B = 5.46, P = 0.035), family income (B = 5.58, P = 0.025), prevention method (B = 10.127, P = 0.000) and action related to infection in the person (B = 12.27, P = 0.047) were the final predictors of sexual self-care score in reproductive age women.

**Conclusion:**

Based on the findings of the study, development of self-care promotion programs for reproductive aged women are necessary in all areas of reproductive health, with a priority for women’s cancer prevention, and focusing on promoting education and related economic assistance. The results of this study can be available to experts and policy makers to design programs to promote sexual self-care in women.

## Introduction

As per the declaration in the International Conference on Population and Development (ICPD) in Cairo, Egypt, sexual and reproductive health (SRH) has been characterized as the state of complete physical, mental, and social well-being in all aspects of the reproductive system along with its functions and processes, but not merely the absence of any disease, weakness, or impairment. Therefore, and SRH approach can help all individuals, in developing and developed nations, to take advantage of SRH services, and broaden their knowledge horizons in this respect [1, 2]. Nevertheless, approximately four billion women of reproductive age (WRA) around the globe do not have access to such services. Against this background, one of the major approaches advocated by the World Health Organization (WHO) is implementing intervention programs to promote self-care in order for public health development [3]. This organization has further delineated self-care as a person's ability to improve and maintain one's health and self-care skills, prevent diseases, and reach adaptation to illnesses and disabilities [3–5]. In addition, self-care interventions can help to improve rights and health [6].

Reflecting on SRH, among the various aspects of personal health that require self-care education, is thus of utmost importance in the public and SRH health intervention programs [7]. As well, self-care interventions can improve SRH and well-being [8], but It seems individuals usually do not meet their needs for improving SRH status and receiving self-care skills from professionals and healthcare providers (HCPs), but mostly prefer to go to pharmacies and purchase modern medications, and the like [9]. In this way, sexual health has been recognized as one of the important health topics, as many women suffer from various sexually transmitted diseases (STDs), such as acquired immunodeficiency syndrome, vaginal infections (or vaginitis), gastrointestinal diseases, skin disorders, etc. On the other hand, unintended pregnancies, domestic violence, plus sexual abuse need to be mentioned in this respect [10, 11].

Even with the significance of self-care in SRH, most studies have been focused on diseases and chronic conditions, such as diabetes mellitus [12, 13], cardiovascular diseases (CVDs) [14], and multiple sclerosis (MS) [15]. Accordingly, there is little information on the sexual self-care status in WRA. While sexual health is an integral part of one's life [1, 16], fear, psychological disorders, false beliefs, etc., bring about disturbances in one's sexual relationships, and then affect the quality of life [16]. Among the definitions presented for sexual health, the WHO has identified it as the ability to get pleasure from one's sexual behaviors and manage them, prevent and control STDs, and deal with psychosexual disorders [17]. To do so, individuals need to practice sexual self-care.

Existing for a long time, the use of self-care products, such as condoms and contraceptive pills, has had profound impacts on individuals' physical and sexual health status. Thus, sexual self-care lies at the heart of self-care for SRH, whose consideration improves gender equality, and contributes significantly to establishing communication and independence. As well, breakthrough technologies have made more self-care products, such as tests, medicines, and self-care programs available [18]. As a result, the presence of intervention programs and strategies based on sexual health promotion along with sex education programs [19, 20] as well as autonomy or personal responsibility for self-care [21], and the installation of posters in pharmacies [9] can be contributing in this regard. However, the interventions for promoting sexual self-care should be further introduced, and then examined by healthcare systems to see whether there is follow-up to meet self-care needs or not [22], and what factors promote sexual self-care?

Given the novelty of sexual self-care, no study on this concept, and the fact that political correctness and cultural sensitivity in a region and the background characteristics of a group should be taken into account for planning in keeping with the same group [23, 24], the present study aimed to explain sexual self-care status and its determinants in the WRA referring to the healthcare centers of Sari, Iran, in 2021.

## Materials and methods

This descriptive cross-sectional study was performed on 400 WRA, referring to the healthcare centers affiliated to Mazandaran University of Medical Sciences, Sari, Iran, in 2021. The inclusion criteria were the samples' willingness to contribute to the study, Iranian nationality, reading and writing literacy, the age range of 18–49, being married and having sex over the last six months, no underlying diseases affecting sexual functioning as per self-reports, taking no medications affecting sexual functioning, no impotence in one's husband, and not being pregnant or breastfeed at the time of study. These individuals were excluded if they were reluctant to continue the study. The sample size was further determined based on the following formula and a pilot study.

$$n = \frac{{z_{{{\raise0.7ex\hbox{$\alpha $} \!\mathord{\left/ {\vphantom {\alpha 2}}\right.\kern-0pt} \!\lower0.7ex\hbox{$2$}}}}^{2} \sigma^{2} }}{{d^{2} }}$$

$$\alpha = .05\quad z = 1.96$$

*σ* is the standard deviation of the sexual performance score, which is equal to 26 based on pilot samples, and d is the estimation accuracy, which is considered equal to 3.

$$\sigma = 26$$

$${\text{d}} = 3$$

$${\text{n}} = 289$$

Consistent with the given formula, 290 samples were required at a minimum. Ultimately, the sample size included 400 people, considering the possibility of 40 percent attrition rate. The sampling method was also of the two-stage systematic random type. For this purpose, four healthcare centers were randomly selected from the three districts in the city of Sari (in line with the zoning), using a random number table. Then, the study samples were randomly selected along with the ratios obtained from each center.

The data collection instrument in this study comprised of the personal-obstetric characteristics and sexual self-care questionnaires for WRA. The Personal-Obstetric Characteristics Questionnaire contained items related to personal and social characteristics as well as obstetric records, such as age, level of education and that of one's husband, medical sciences education, employment status and that of one's husband, household income, duration of marriage, current contraceptive method, gravida, number of abortions, sources of sex information, infections over the past month, action in case of infections, information about breast exams, as well as information about HIV test and HPV vaccination history. The validity of the Personal-Obstetric Characteristics Questionnaire items was examined using qualitative content validity. For this purpose, the questionnaire was provided to 15 Sexual and Reproductive health professionals, and their opinions were applied.

The next questionnaire was a researcher-made one to evaluate sexual self-care. The female sexual health self-care (FSHS) questionnaire. This questionnaire was designed using an extensive literature review on the valid references [1, 25–31] and based on the four steps Waltz for tool development [32]. The items of the FSHS were scored with a 5-point Likert including never (1), rarely (2), sometimes (3), often (4) and always (5) points. The psychometric characteristics of this questionnaire showed that the total validity index of the instrument was 0.93 and the validity ratio of the entire instrument was 0.96. The relative reliability of the whole tool was also shown with Cronbach's alpha of 0.94. The stability of the tool was also shown by the ICC in the whole tool of 0.97 with a confidence interval of 0.94–0.98.

The final version of FSHS was designed with 40 items in four factors including Prevention of sexually transmitted diseases and genital infection (14 questions) minimum score 14 and maximum score 70, prevention of women's cancers (7 questions) maximum score 35 and minimum score 7, prevention of unwanted pregnancy (6 questions) the maximum score is 30 and the minimum is 6, and in the sexual health promotion subscale (13 questions), the maximum score is 65 and the minimum score is 13. It should be noted that the average of the total tool (40 questions) is a maximum of 200 and a minimum of 40. In order to better understand scoring and its comparability, the scores of each factor were converted into zero to hundred scores, and as a result, the score of the instrument was considered zero to hundred. The higher the score, the more sexual self-care.

Upon receiving approval from the Ethics Committee of the School of Nursing and Midwifery at Shahid Beheshti University of Medical Sciences, Tehran, Iran (Code of Ethics: IR.SBMU.PHARMACY.REC.1399.200), the researcher attended the selected healthcare centers, obtained permission from their officials, negotiated with the family health unit staff to cooperate in the research project, and described the main objective of the study and its sampling procedure. Afterward, a list of the WRA in the selected centers was prepared, and two-stage systematic random sampling was operated. After sample selection, the researcher contacted them, provided them with the necessary explanations, and asked them to take part in the project. After obtaining verbal and written consent, they were included in the study. Given the spread of the coronavirus disease 2019 (COVID-19) pandemic and the risks of its transmission as well as the fewer visits to the healthcare centers by these women, electronic sampling was implemented by the Google platform, based on some fixed responses.

To complete the questionnaires, their electronic forms were sent to the samples through WhatsApp Messenger. The first item was related to agreement with participating in the study based on a fixed response, so the subsequent items could come into view if it was approved. If the samples did not have access to smartphones, they could come in person and fill the questionnaires on paper. Finally, some statistics, such as t-test, Chi-square test, analysis of variance (ANOVA), Mann–Whitney U test, and linear regression analysis were implemented, using the SPSS software package (ver. 22.0), to analyze the data.

## Results

Based on the study findings, the WRA's mean age was 30.45 ± 5.53. Considering the current contraceptive methods practiced by the samples, the most frequent one was condom use (63.3%). Other findings associated with the personal and social characteristics as well as the obstetric records are illustrated in Table 1.

**Table 1:** Socio-demographic and obstetrics information of 400 WAR

Variable	Mean ± SD/N (%)
Age (years)	30.45 ± 5.53
Duration of marriage (years)	6.81 ± 4.83
*Education level*	
Diploma	51 (13)
BS	234 (58.2)
MS	103 (25.5)
Ph.D.	12 (3.3)
*Spouse’s educational level*	
Middle school	12 (3)
Diploma	85 (21.3)
BS	193 (48.3)
MS	90 (22.5)
Ph.D.	20 (5)
*Education related to medical health*	
Yes	80 (20)
No	320 (80)
*Employment status*	
Housewife	145 (36.3)
Employee	206 (51.4)
Student	33 (8.3)
looking for work	16 (4)
*Spouse’s employment status*	
Employee	162 (40.5)
Worker	18 (4.5)
Own job	204 (51)
Student	6 (1.5)
Looking for work	10 (2.5)
*Economic status*	
Satisfied	67 (16.7)
Intermediate	277 (69.3)
Dissatisfied	56 (14)
*Contraception method*	
Hormonal	65 (16.1)
Condom	145 (36.3)
Natural birth control	118 (29.5)
Tubectomy and Vasectomy	7 (1.8)
No method	65 (16.3)
*History of HIV testing*	
Yes	95 (23.8)
No	305 (76.3)
*History of HPV vaccination*	
Yes	17 (3.4)
No	383 (95.8)
*A source of sexual information*	
Book	35 (8.8)
Web-site	67 (16.8)
Social media	231 (57.8)
Health center	28 (7)
Other	39 (9.6)
*Age at the time of the first breast examination*	
Without examination	223 (55.8)
18–25 years	93 (23.1)
26–30 years	48 (12)
31 years and more	36 (9.1)
*Number of breast examinations*	
Just 1	78 (19)
2–3 times	58 (13.8)
4 times and more	41 (9.8)
*Action if there are signs of infection*	
Without treatment	230 (57.5)
Previous medication	39 (9.8)
Doctor or midwife	101 (25.3)
Online visit and herbal medicine	30 (7.4)

After implementing the sexual self-care questionnaire for WRA, the sexual self-care mean value in these women was determined. The total mean score of sexual self-care in the WRA here was 70.66 ± 12.52. In addition, the prevention of women's cancers and the prevention of unintended pregnancies obtained the lowest and highest scores, respectively (Table 2).

**Table 2:** Mean and standard deviation of sexual self-care score based on hundreds of 400 WAR

Variable	Mean	Standard deviation
Prevention of women's cancers	61.31	22.35
Promotion of sexual health	69.01	15.65
Prevention of sexually transmitted diseases and genital infections	74.12	16.36
Prevention of unwanted pregnancy	77.12	13.49
The total score of the sexual self-care	70.66	12.52

The study results demonstrated a significant relationship between the mean and standard deviation (SD) values of sexual self-care and women's age, duration of marriage, education, husband's education, household income, and medical sciences education. However, no significant relationship was observed between women's employment status and that of their husband and sexual self-care (Table 3). As per the t-test results, the mean score of sexual self-care in the group with the history of HIV testing was higher than that in the ones that did not mention this history, so this difference was statistically significant. Moreover, the mean value of sexual self-care in the group with the history of HPV vaccination was higher than that in the ones that did not receive this vaccine, so this difference was statistically significant. A statistically significant relationship was further found between the different sources of sex information tapped by these women and the mean score of sexual self-care. Based on the follow-up test of the individuals whose sources of sex information were their friends, the sexual self-care mean score among them was lower than other samples. In addition, the study results showed that the mean score of sexual self-care in women who received breast exams for the first time at the age of 30 was lower than that of the cases with such exams at a younger age, so this difference was statistically significant. Besides, a significant relationship was found between the number of breast exams and the sexual self-care mean score. Based on the follow-up test, the samples who received only one exam reported the lowest self-care mean score, compared to other people with more breast exams. Comparing the mean and SD values of the sexual self-care variable also revealed a statistically significant relationship between the current contraceptive methods and the mean score of sexual self-care and between the action in case of infections and the mean value of sexual self-care. Accordingly, individuals who used condoms had a higher mean score of sexual self-care, and those taking herbal medicines in case of infections received the lowest mean value (Table 3).

**Table 3:** The relationship between the mean score of sexual self-care and socio-demographic and obstetrics variable of 400 WAR factors

Variable	Test statistic	P value
Age	r = − 0.56	0.021*
Duration of marriage	r = − 0.224	0.042*
Education level	F = 2.48	0.02*
Spouse’s educational level	F = 2.95	0.02*
Education related to medical health	F = 0.006	0.001*
Employment status	F = 1.20	0.30
Spouse’s Employment status	F = 1.39	0.23
Economic status	F = 1.59	0.19
Contraception method	F = 7.67	0.000*
History of HIV testing	T = 3.07	0.002*
History of HPV vaccination	T = 3.74	0.001*
A source of sexual information	F = 2.30	0.026*
Age at the time of the first breast examination	F = 3.93	0.002*
Number of breast examinations	F = 4.00	0.008*
Action if there are signs of infection	F = 2.25	0.04*

*r* Pearson correlation coefficient, *F* one-way ANOVA, *t *independent sample t-test

*Significant

As well, multiple Linear Regression analysis was performed in order to determine the predictive level of the variables. In view of that, the variables with the significance level of less than 0.2 (p < 0.2) were imported into the primary multiple Linear Regression model, thus ensuring that all potential variables predicted were included. Based on the final Linear Regression model, medical sciences education (β = 5.46, p = 0.035), household income (β = 5.58, p = 0.025), contraceptive methods (β = 10.127, p = 0.000), and action in case of infections (β = 12.27, p = 0.047) were the main predictors of sexual self-care mean score in the WRA (Table 4). In cases with medical sciences education, compared to those having irrelevant education, the mean score of sexual self-care elevated by 5.460 on condition that all the variables remained constant. In the women having sufficient household income, compared to the ones who had more and above sufficient income, the self-care mean value dropped by 5.587 with the stipulation that all the variables were constant. The sexual self-care mean score also increased by 10.127 for the individuals who used condoms as a contraceptive method, compared to other methods, on condition that all the variables remained constant. Among the women with the history of infection over one month, the sexual self-care mean value diminished by 12.272, compared to those who had online visits or took herbal medicines with the stipulation that all the variables were constant.

**Table 4:** Final predictors of sexual self-care score of WAR according to multiple linear regression analysis

Variable	Domain	B	SE	P value	95% CI
Age		− 0.341	0.165	0.87	(− 0.001,0.764)
Duration of marriage		− 0.444	0.216	0.040*	(− 0.020, − 0.086)
Education level^b^	Diploma	− 4.640	3.537	0.190	(− 11.574, 2.293)
	BS	0.717	2.214	0.746	(− 3.624, 5.058)
	MS AND Ph.D.^a^	–	–	–	–
Spouse’s educational level^c^	Diploma and less	− 0.098	2.865	0.973	(− 5.714, 5.519)
	BS	1.013	2.246	0.652	(− 3.391, 5.417)
	MS AND Ph.D.^a^	–	–	–	–
Education related to medical health	Yes	5.460	2.596	0.035*	(0.371, 10.550)
	No^a^	–	–	–	–
Economic status^d^	Dissatisfied	− 5.325	3.357	0.113	(− 11.905, 1.255)
	Intermediate	− 5.587	2.487	0.025	(− 10.463, − 0.712)
	Satisfied^a^	–	–	–	–
Contraception method	Hormonal	− 3.952	4.193	0.346	(− 12.171, 4.267)
	Condom	10.127	2.735	0.000*	(4.765, 15,490)
	Natural birth control	− 3.876	2.784	0.164	(− 9.334, 1.582)
	Tubectomy and vasectomy	− 0.990	3.873	0.798	(− 8.583, 6.602)
	No method^a^	–	–	–	–
History of HIV testing	Yes	3.655	1.977	0.065	(− 0.221, 7.530)
	No^a^	–	–	–	–
History of HPV vaccination	Yes	6.832	4.627	0.140	(− 2.239, 15.902)
	No^a^	–	–	–	–
A source of sexual information	Book	7.736	4.290	0.071	(− 0.673, 16,145)
	Web-site	2.328	3.676	0.527	(− 4.878, 9.534)
	Social media	3.288	3.138	0.293	(− 2.844, 9.421)
	Health center	8.081	4.487	0.072	(− 0.715, 16.877)
	Other^a^	–	–	–	–
Age at the time of the first breast examination	Without examination	− 7.213	4.887	0.140	(− 16.791, 2.366)
	18–25 years	− 2.962	3.894	0.447	(− 10.594, 4.670)
	26–30 years	− 5.656	4.267	0.185	(− 14.022, 2.711)
	31 years and more^a^	–	–	–	–
Number of breast examinations^e^	Just 1	− 1.205	4.986	0.809	(− 10.979,8.569)
	2–3 times	− 0.434	3.905	0.912	(− 8.088, 7.220)
	4 times and more^a^	–	–	–	–
Action if there are signs of infection	Previous medication	− 3.912	3.118	0.210	(− 10.025, 2.201)
	Doctors or midwifes	2.604	2.260	0.249	(− 1.827, 7.035)
	Online visit and herbal medicine	− 12.272	6.173	0.047*	(− 24.373, − 0.172)
	Without treatment^a^	–	–	–	–

*B* unstandardized regression coefficient, *SE* standard error, *CI* confidence interval

*Significant

^a^Baseline

^b^Classification of Education: 1. Diploma and under diploma, 2 BS, 3. MS AND Ph.D.

^c^Classification of Spouse’s educational level: 1. Diploma and under diploma, 2 BS, 3. MS AND Ph.D.

^d^Classification of Economic status: 1. Dissatisfied, 2. Intermediate, 3. Satisfied

^e^Classification of number of breast examination: 1. Just 1, 2. 2–3 times. 3. 4 times and more

## Discussion

This quantitative study aimed to explain sexual self-care status and its determinants in the WRA, referring to healthcare centers of Sari, Iran, in 2021. Based on the Sexual Self-Care Questionnaire for WRA, the total mean value of sexual self-care in the women recruited in this study was 70.66 ± 12.52. This questionnaire was further designed, and its psychometric properties were evaluated for the first time in this study; therefore, there was no similar survey in the field of determining the mean score of sexual self-care in WRA.

With reference to the study findings, there was a statistically significant difference between the women's age the sexual self-care mean score. Following the increase in age, the mean score of self-care in these individuals decreased. The results further revealed that sexual functioning declined with age in these women [33], as one of the reasons for the fall in the mean value of sexual self-care. Of note, older women in some cultures may feel that they must hide their sexuality in order to match up with many social norms [34], making them less likely to seek measures to improve their sexual self-care skills. The older people are thus at greater risk of exposure to STDs, and above all, they are physiologically more vulnerable to infections. For example, postmenopausal changes in the vaginal epithelium can reduce innate defense mechanisms against infections. The immune system functioning also deteriorates throughout life, increasing vulnerability to HIV infection [34]. As a result, more attention must be paid to sexual self-care education for women as they age.

Moreover, the study findings showed a significant relationship between the women's education, their husband's education, and sexual self-care. The mean score of sexual self-care among the women who themselves or their husbands had high school diplomas was lower than other individuals. Accordingly, one of the most important factors promoting sexual self-care in women is providing information and raising awareness. For example, women try to treat themselves with various information they get from their surroundings [9]. Therefore, adopting an approach or developing a strategy to empower women and broaden their knowledge horizons can boost their SRH status [2]. In line with the present study, a survey in Turkey similarly found a significant relationship between the low levels of education and the poor sexual functioning in women [35]. Accordingly, women's independence can play a leading role in their SRH self-care. This means that women should have the ability to hear and speak, make decisions about one's life, and ultimately enjoy a life free from violence, fear, and terror [36]. This is not possible except by fostering women's awareness of their rights and empowering them. In addition, various studies have reported that the Pap test is more common in the individuals with higher education and sufficient household income [37, 38], which consequently improves sexual self-care.

Based on the study findings, the mean score of sexual self-care in the group with the history of HIV testing was significantly higher than the ones that did not report it. Pre- and post-test counseling could be thus one of the reasons for the upward trend in the mean value of sexual self-care in women with the history of HIV testing. In this line, one survey had found that HIV counseling after a positive test result could increase condom use [39]. Broadening knowledge and information is accordingly of importance in order to boost sexual self-care, because it changes a person's behavior and elevates their ability and self-confidence, which lead to their self-efficacy, as a protective factor against high-risk sexual behaviors, such as AIDS prevention in sexual relationships [40, 41]. In addition, conducting educational interventions appropriately can expand knowledge, provoke positive changes in attitudes, and improve the performance of vulnerable women against HIV and AIDS [31]. In this respect, one study on women with HIV had demonstrated that self-care and social support could redouble health-related quality of life in this group [42].

Likewise, the mean score of sexual self-care in the group that had the history of HPV vaccination was significantly higher than the ones that did not mention it. HPV infection is thus one of the most common STDs worldwide [43], and the major infection complaint among young and sexually active people, so that over 75% of sexually active individuals experience it during their lifetime [44]. The cause of more than 90% of cervical cancers is previous infection with HPV, which is caused by sexual contact with an infected person [45]. In general, vaccination is one of the effective ways to prevent the stage-one cervical cancer. Of note, reducing the incidence rate of cervical cancer and its lesions is one of the main objectives of HPV vaccine production [46]. According to a review study, an effective technique for promoting self-care practices in WRA was preventive and screening behaviors, such as tests for women's cancers and STDs [47]. Increasing awareness about the spread of HPV and its modes of transmission, as well as vaccination against this virus, can thus lead to an improvement in disease control and minimize the consequences [48]. Correct knowledge about the virus epidemiology can further help adopt protective behaviors, such as condom use, thereby reducing the risk of infection. This highlights the importance of strengthening the knowledge foundations about HPV. Even if increased knowledge alone is not protective enough, having accurate information about HPV can influence health promotion attitudes, in so doing improving sexual health status by health-seeking practices [49].

A statistically significant relationship was also observed between the sources of sex information utilized by women and the sexual self-care mean score. In this regard, women whose friends were the main sources of sex information had the lowest sexual self-care mean values. Empowering women to search for sex information can be thus effective in promoting sexual self-care [50]. Since a significant relationship has been reported between health literacy and self-care in women based on previous research [51], it is essential to provide them with health-related information in the correct form and through correct channels. In this line, a survey had indicated that young people receiving information from their parents were more likely to start sexual activities later than their peers who had access to sex information from their friends. However, evidence shows that some young people prefer to receive sexual health information from health professionals [52]. A study had further reported that the main sources of information about HPV vaccination were magazines and books (33%), TV programs (26.7%), and gynecologists (23.3%), respectively [53]. In a survey in Iran, 70% of the participants had also mentioned HCPs as the most important sources of information about family planning methods [54]. Therefore, it is of utmost importance that all professionals and HCPs become aware of SRH to provide the basic advice to increase sexual self-care.

The study findings correspondingly showed that the mean score of sexual self-care was lower in women who received their first breast exam at an older age (namely, 40 and older), and this difference was statistically significant. In addition, a significant relationship was found between the number of breast exams and the mean score of sexual self-care. Accordingly, the individuals who had received a breast exam one time reported the lowest mean score of sexual self-care. Of note, one of the most common women's cancers is breast cancer, whose mortality rate can be reduced with timely diagnosis and treatment. A review study had thus reported that Iranian women's awareness of breast cancer prevention behaviors, such as breast self-exams, clinical breast exams, and mammography was very low [55]. Therefore, providing public education and encouragement for women, especially the non-working, married, low-educated, and young ones, as well as preparing a suitable place to perform breast self-exams in healthcare centers seem necessary [56].

Based on the study findings, the total mean score of sexual self-care in the WRA participating in this study was 70.66 ± 12.52. The questionnaire for this purpose was designed and its psychometric properties were evaluated for the first time here; therefore, there was no similar study in the field of determining the mean score of sexual self-care in these individuals. The prevention of women's cancers also received the lowest amount of self-care. In this line, a survey had revealed that the performance of women in the field of cancer screening was often low, although the general knowledge of cervical cancer was relatively high. In addition, this study showed unfavorable ideas and beliefs about cervical cancer among women, which required more education to fill the existing gaps between women's knowledge and practice to prevent cancer [57]. With reference to the study findings, breast self-exam in the WRA was low. Meanwhile, a study had indicated the effectiveness of self-care education programs based on the trans-theoretical model in women referring to healthcare centers to perform breast self-exams, thereby improving their health status [58]. On the other hand, self-care measures could play an important role in minimizing disease symptoms, augmenting physical and mental conditions, and ultimately promoting the quality of life of women with cancer, which denoted the necessity of self-care education for women [59, 60].

The study findings additionally revealed that women received the lowest mean score in the field of cancer prevention. In this regard, HPV testing had the lowest mean value. Although more women may have the Pap test, the combination of HPV testing with the Pap test is still not seen in many women, because HPV test positivity is linked with some adverse psychosocial outcomes, mainly due to the sexually transmitted nature of the virus and its association with cervical cancer [61]. In this respect, a study had reported that HPV testing had the potential to cause psychosocial harm to women, their partners, and their families [62]. As a result, HPV testing should be accompanied by extensive health education to inform women and de-stigmatize the infection to ensure that any adverse effects on women's health status and possible psychological effects are minimized [61].

Based on the study findings, the use of condoms in all sexual relationships received the lowest score in the field of the prevention of STDs. With regard to unintended pregnancy prevention, the WRA obtained a low mean score for using condoms if they had a hormonal contraceptive method, because condoms were viewed only as a means of preventing pregnancy but not STDs and infections. Other studies had also shown that the use of condoms as a means of preventing STDs was still low [63, 64]. This is despite the fact that it is possible to use a two-method approach, i.e., a method for preventing pregnancy (such as taking pills or having injections, etc.) and a method used to prevent STDs (including using condoms), based on each society and the prevalence rate of STDs and infections. In addition, promoting the use of condoms to prevent pregnancy, instead of (or in addition to) preventing STDs, can lead to more use of condoms, and thus reduce the rate of infection in society [65].

According to the RA results in this study, medical sciences education was one of the predictors of the total mean score of sexual self-care in the WRA. Considering the familiarity of the women having medical sciences education with the anatomy and physiology of the female reproductive system and the factors affecting the sexual cycle, the higher mean scores of sexual self-care in these individuals was justified. In addition, education could provide empowerment in the direction of the ability to change one's lifestyle and the development of communication skills and negotiations to resolve conflicts, which can be useful in the sexual and personal health of women as well [5]. In this regard, a study had reported that the inadequate information provided by the health system, insufficient knowledge of sexual issues, and the lack of academic education were among the factors that could reduce the promotion of sexual health in people [66]. Another study had also proved that education through the educational systems for adolescent girls could empower them, and thus improve their SRH status [67]. In fact, higher levels of knowledge were associated with greater sexual assertiveness and confidence in using condoms among women [68]. Therefore, sex education is related to the effect of knowledge on sexual health, prevention of STDs, and improvement of personal and interpersonal communication to promote sexual health [40, 41, 69, 70].

Another predictor of the total mean score of sexual self-care in the WRA was household income. Other studies had further established the relationship between socioeconomic status and self-care skills in patients with heart failure [71]. It seems that socioeconomic status and social support are related to sexual functioning in WRA [72]. An intervention study had accordingly revealed that the regular use of contraceptives and condoms were associated with one's employment status and family's economic status, respectively [73]. In other words, women's income could increase their access to family planning services [74], as a factor to improve their sexual self-care status. On the other hand, economic status could shape other aspects of sexual self-care. For example, economic factors and imbalance in gender relations and power in sexual negotiations could be among the main factors affecting women's vulnerability to HIV infection. Inequality in socioeconomic opportunities was further related to the higher vulnerability of young people to HIV and AIDS [75].

According to the RA results here, contraceptive methods could predict the total mean score of sexual self-care in the WRA. According to the study findings, the mean score of sexual self-care was higher among people whose contraceptive method was condoms. As well, the most common contraceptive method used by women in a study in Urmia, Iran, was natural contraception. In relation to this study, the level of sexual satisfaction in the WRA was different based on the contraceptive method utilized. In this respect, the individuals who mentioned natural contraception and condoms had the highest sexual satisfaction. The lowest sexual satisfaction was further associated with women who mentioned taking contraceptive pills as a priority. In addition, those with hormonal injections had the highest rate of sexual dysfunction [76]. The findings of a study in Iran had also revealed that contraceptive methods could affect women's sexual satisfaction [77]. In general, sexual health is an important public health issue in all age groups. Condom use has been further promoted as one way to minimize and prevent the unwanted consequences of sexual behaviors, but its overall use remains low [64]. Sexual health promotion programs can accordingly improve women's knowledge of the effectiveness of condoms to prevent pregnancy, followed by testing to follow up and prevent STDs [78].

Furthermore, the study results revealed that the action in case of infections was one of the predictors of sexual self-care in the WRA. Accordingly, women who took herbal medicines in case of infections obtained a lower self-care mean score. Vaginal infections are thus one of the most common conditions that women suffer from due to poor awareness. In this regard, the study findings showed that the WRA with vaginal infections needed more education about the genital tract infections and the related self-care processes [79]. In fact, one of the most important educational needs in the WRA regarding vaginal infections is sexual self-care [80]. Therefore, the use of educational interventions, such as the intervention based on the PRECEDE-PROCEED model, having an effect on awareness, raising attitudes, performing health behaviors, and bolstering reinforcing and enabling factors, can improve women's health regarding the prevention of infections [81]. Based on a study, factors related to vaginal infection prevention methods were education, place of residence, and perceived susceptibility. Those who took fewer measures to prevent vaginal infections were thus women with a low level of education and living in rural areas. According to the Health Belief Model, women who were less susceptible to vaginal infections were less likely to take preventive measures [82]. As a result, there is a need to provide sufficient information to the WRA in the field of sexual self-care for vaginal infections, in order to prevent infections and increase the number of women's visits to healthcare centers and HCPs in case of presenting symptoms.

This study was one part of a multi-stage comprehensive research project. Considering the vital role of sexual self-care in women's health and the impact of some determinants, such as education, household income, contraceptive methods, and action in case of infections on sexual self-care, it was suggested to implicate the results in the design, implementation, and evaluation of sexual self-care promotion interventions and the related programs for WRA.

## Strengths and limitations

Designing a questionnaire devoted to sexual self-care in WRA, which was not available before, was one of the strengths of this study. Among the limitations, arising here, was the descriptive research design that could merely show the relationship between the study variables, but not the cause-and-effect relationships. In addition, sampling during the COVID-19 pandemic and the subsequent problems of this crisis might have influenced the participants' responses. The use of women's self-reports was another limitation, which could give rise to some errors. Using electronic sampling is another limitation of our study. Also, the exploratory factor analysis (EFA) or Confirmatory Factor Analysis (CFA) were not performed because the self-care dimensions and items are known, and EFA or CFA are compulsory for exploring or confirming the construct of a valid instrument [32]. But EFA or CFA are possible and suggested to be performed in future studies.

## Conclusion

The present study established that the sexual self-care status in the WRA could be predicted by medical sciences education, household income, contraceptive methods, and action in case of infections. Therefore, professionals and HCPs should be aware of the relationship between these determinants, and take necessary measures to promote sexual self-care skills among WRA. The improvement of socioeconomic factors, such as education and household income, is also associated with higher mean scores of sexual self-care, which should be considered by policymakers. Based on the findings of the study, development of self-care promotion programs for reproductive aged women are necessary in all areas of reproductive health, with a priority for women’s cancer prevention, and focusing on promoting education and related economic assistance. In the end, the professionals and policymakers in the health system should be supplied with the study results to develop intervention programs in order for promoting sexual self-care in WRA.

## Data Availability

All data generated or analyzed during this study are available upon request from the corresponding author. Any additional data/files may be obtained from the corresponding author.

## Abbreviations

SRH: Sexual and reproductive health
AIDS: Acquired immunodeficiency syndrome
HIV: Human Immunodeficiency Virus
HPV: Human papilloma virus
WRA: Women of reproductive age
WHO: World Health Organization
STD: Sexually transmitted diseases
HCPs: Healthcare providers

## References

[1] World Health Organization. WHO consolidated guideline on self-care interventions for health: sexual and reproductive health and rights. 2019. 31334932

[2] Swatzyna RJ, Pillai VK (2013). The effects of disaster on women's reproductive health in developing countries. Global J Health Sci.

[3] World Health Organization. Economic and financing considerations of self-care interventions for sexual and reproductive health and rights: United Nations University Centre for policy research, 2–3 April 2019, New York, United States of America: summary report. World Health Organization, 2019.

[4] Ma J. Interacting with health information for self-care: an exploratory study of undergraduate students' health information literacy. 2014.

[5] Behboodi Moghadam Z, Rezaei E, Yalegonbadi K, Montazeri A, Arzaqi SM, Tavakol Z (2015). The effect of sexual health education program on women sexual function in Iran. J Res Health Sci.

[6] Ferguson L, Fried S, Matsaseng T, Ravindran S, Gruskin S (2019). Human rights and legal dimensions of self care interventions for sexual and reproductive health. BMJ.

[7] Davis LM, Choudhury S, Erausquin JT, Withers M (2018). Women’s partner relationships and reproductive and sexual health in Lusaka, Zambia. Global perspectives on women's sexual and reproductive health across the lifecourse.

[8] Logie CH, Berry I, Ferguson L, Malama K, Donkers H, Narasimhan M (2022). Uptake and provision of self-care interventions for sexual and reproductive health: findings from a global values and preferences survey. Sex Reprod Health Matters.

[9] Hardon A, Pell C, Taqueban E, Narasimhan M (2019). Sexual and reproductive self care among women and girls: insights from ethnographic studies. BMJ.

[10] Albuquerque AFLL, Pinheiro AKB, Linhares FMP, Guedes TG (2016). Technology for self-care for ostomized women's sexual and reproductive health. Rev Bras Enferm.

[11] American College of Obstetricians AND Gynecologists. Your sexual health 2021. Available from: https://www.acog.org/womens-health/faqs/your-sexual-health.

[12] Wangberg SC (2008). An Internet-based diabetes self-care intervention tailored to self-efficacy. Health Educ Res.

[13] Nwasuruba C, Khan M, Egede LE (2007). Racial/ethnic differences in multiple self-care behaviors in adults with diabetes. J Gen Intern Med.

[14] Holden RJ, Schubert CC, Mickelson RS (2015). The patient work system: an analysis of self-care performance barriers among elderly heart failure patients and their informal caregivers. Appl Ergon.

[15] Masoudi R, Mohammadi E, Nabavi SM, Ahmadi F (2008). The effect of Orem based self-care program on physical quality of life in multiple sclerosis patients. J Shahrekord Univ Med Sci.

[16] Simbar M, Azin SA (2017). The development and validation of sexual health education needs assessment questionnaire of Iranian engaged couples. Galen Med J.

[17] Edwards WM, Coleman E (2004). Defining sexual health: a descriptive overview. Arch Sex Behav.

[18] Remme M, Narasimhan M, Wilson D, Ali M, Vijayasingham L, Ghani F (2019). Self care interventions for sexual and reproductive health and rights: costs, benefits, and financing. BMJ.

[19] Braeken D, Cardinal M (2008). Comprehensive sexuality education as a means of promoting sexual health. Int J Sex Health.

[20] Khalesi ZB, Simbar M, Azin SA, Zayeri F (2016). Public sexual health promotion interventions and strategies: a qualitative study. Electron Phys.

[21] Murray SJ (2007). Care and the self: biotechnology, reproduction, and the good life. Philos Ethics Humanit Med.

[22] World Health Organization. United Nations University. Economic and financing considerations of self-care interventions for sexual and reproductive health and rights. 2020:1–19.

[23] World Health Organization (2019). Adopting self-care interventions for sexual and reproductive health in the Eastern Mediterranean Region. East Mediterr Health J.

[24] Fang X, Li X, Yang H, Hong Y, Stanton B, Zhao R (2008). Can variation in HIV/STD-related risk be explained by individual SES? Findings from female sex workers in a rural Chinese county. Health Care Women Int.

[25] American College of Obstetricians AND Gynecologists. Breast cancer risk assessment and screening in average-risk women. Pract Bull. 2017;(179):1–16.

[26] Khazaee-Pool M, Majlessi F, Montazeri A, Pashaei T, Gholami A, Ponnet K (2016). Development and psychometric testing of a new instrument to measure factors influencing women’s breast cancer prevention behaviors (ASSISTS). BMC Womens Health.

[27] Saulle R, Miccoli S, Unim B, Semyonov L, Giraldi G, De Vito E (2013). Validation of a questionnaire for young women to assess knowledge, attitudes and behaviors towards cervical screening and vaccination against HPV: survey among an Italian sample. Epidemiol Biostat Public Health.

[28] Gil-Llario M, Ruiz-Palomino E, Morell-Mengual V, Giménez-García C, Ballester-Arnal R (2019). Validation of the AIDS prevention questionnaire: a brief self-report instrument to assess risk of HIV infection and guide behavioral change. AIDS Behav.

[29] Workowski KA (2021). Sexually transmitted infections treatment guidelines, 2021. CDC Recomm Rep.

[30] Martínez-Urquijo A, Postigo Á, Cuesta M, Fernández-Álvarez MdM, Martín-Payo R (2021). Development and validation of the MARA scale in Spanish to assess knowledge and perceived risks and barriers relating to breast cancer prevention. Cancer Causes Control.

[31] Jacobson JD. Women and sexual problems 2020. Available from: https://medlineplus.gov/ency/patientinstructions/000663.htm.

[32] Ebadi A, Sharif Nia H, Zareiyan A, Zarshenas L. Instrument development in health sciences, 2nd ed. Jamee Negar; 2019.

[33] Lee DM, Nazroo J, O’Connor DB, Blake M, Pendleton N (2016). Sexual health and well-being among older men and women in England: findings from the English longitudinal study of ageing. Arch Sex Behav.

[34] Lusti-Narasimhan M, Beard JR (2013). Sexual health in older women. Bull World Health Organ.

[35] Aslan E, Beji NK, Gungor I, Kadioglu A, Dikencik BK (2008). Prevalence and risk factors for low sexual function in women: a study of 1009 women in an outpatient clinic of a university hospital in Istanbul. J Sex Med.

[36] Lupton D (2015). Quantified sex: a critical analysis of sexual and reproductive self-tracking using apps. Cult Health Sex.

[37] Sheikhizadeh S, Ahmadipour H (2016). Self-care activities among women referred to health care centers in Kerman. J Health Based Res.

[38] Saberi F, Sadat Z, Abedzadeh M (2012). Factors associated with cervical cancer screening and its barriers among women: Kashan. Iran Payesh (Health Monit).

[39] Shapiro K, Ray S (2007). Sexual health for people living with HIV. Reprod Health Matters.

[40] Assarzadeh R, Bostani Khalesi Z, Jafarzadeh-Kenarsari F (2019). Sexual self-efficacy and associated factors: a review. Shiraz E-Med J.

[41] Rabie N, Fasihi Harandi T, Qorbani M (2017). A survey on the effect of group-discussion to HIV prevention in self-care vulnerable women, in Karaj in 2014–2015. Iran J Health Educ Health Promot.

[42] Gielen AC, McDonnell K, Wu AW, O’Campo P, Faden R (2001). Quality of life among women living with HIV: the importance violence, social support, and self care behaviors. Soc Sci Med.

[43] Serrano B, Brotons M, Bosch FX, Bruni L (2018). Epidemiology and burden of HPV-related disease. Best Pract Res Clin Obstet Gynaecol.

[44] Weaver BA (2006). Epidemiology and natural history of genital human papillomavirus infection. J Am Osteopat Assoc.

[45] American College of Obstetricians and Gynecologists (2003). Cervical cytology screening. Obstet Gynecol.

[46] Lotfi R, Kamrani MA (2015). Knowledge about cervical cancer, human papilloma virus and attitude towards acceptance of vaccination among female students. Payesh (Health Monit).

[47] Bozorgi N, Shahhosseini Z (2017). The effective techniques to promote self-care behaviors in women of reproductive age: a narrative review study. NHJ.

[48] Hasanzadeh Mofrad M, Jedi L, Ahmadi S (2016). The role of human papilloma virus (HPV) vaccines in prevention of cervical cancer, review article. Iran J Obstet Gynecol Infertil.

[49] Swenson RR, Rizzo CJ, Brown LK, Vanable PA, Carey MP, Valois RF (2010). HIV knowledge and its contribution to sexual health behaviors of low-income African American adolescents. J Natl Med Assoc.

[50] Moghasemi S, Ozgoli G, Simbar M, Nasiri M (2018). Middle-aged Iranian women's accounts of their sexual health care practices: a conventional content analysis. Int Perspect Sex Reprod Health.

[51] Tira M, Rahvar M (2018). The influence of health literacy on self-care in women aged between 17–69 years, who visited municipality cultural centers. J Health Lit.

[52] Straw F, Porter C (2012). Sexual health and contraception. Arch Dis Child-Educ Pract.

[53] Saulle R, Miccoli S, Unim B, Semyonov L, Giraldi G, De Vito E (2013). Validation of a questionnaire for young women to assess knowledge, attitudes and behaviors towards cervical screening and vaccination against HPV: survey among an Italian sample. Epidemiol Biostat Public Health.

[54] Modabber M, Pouresmaeil M, Soltani M (2017). Effects of group discussion on knowledge, attitude and practice of women using natural family planning methods in Alborz city, Qazvin. J Health.

[55] Naghibi SA, Shojaizadeh D, Yazdani CJ, Montazeri A (2015). Breast cancer preventive behaviors among Iranian women: a systematic review. Payesh (Health Monit).

[56] Mokhtari L, Khorami Markani A, Habibpoor Z (2014). Correlation between health beliefs and breast cancer early detection behaviors among females referring to health centers in Khoy city. Iran J Health Promot Manag.

[57] Mukama T, Ndejjo R, Musabyimana A, Halage AA, Musoke D (2017). Women’s knowledge and attitudes towards cervical cancer prevention: a cross sectional study in Eastern Uganda. BMC Womens Health.

[58] Ghahremani L, Mousavi Z, Kaveh MH, Ghaem H (2016). Self-care education programs based on a trans-theoretical model in women referring to health centers: breast self-examination behavior in Iran. Asian Pac J Cancer Prev APJCP.

[59] Wang Z, Yin G, Jia R (2019). Impacts of self-care education on adverse events and mental health related quality of life in breast cancer patients under chemotherapy. Complement Ther Med.

[60] Chin C-H, Tseng L-M, Chao T-C, Wang T-J, Wu S-F, Liang S-Y (2021). Self-care as a mediator between symptom-management self-efficacy and quality of life in women with breast cancer. PLoS ONE.

[61] McCaffery K, Waller J, Nazroo J, Wardle J (2006). Social and psychological impact of HPV testing in cervical screening: a qualitative study. Sex Transm Infect.

[62] McCaffery K, Forrest S, Waller J, Desai M, Szarewski A, Wardle J (2003). Attitudes towards HPV testing: a qualitative study of beliefs among Indian, Pakistani, African-Caribbean and white British women in the UK. Br J Cancer.

[63] Sanders SA, Yarber WL, Kaufman EL, Crosby RA, Graham CA, Milhausen RR (2012). Condom use errors and problems: a global view. Sexual Health.

[64] Pinyopornpanish K, Thanamee S, Jiraporncharoen W, Thaikla K, McDonald J, Aramrattana A (2017). Sexual health, risky sexual behavior and condom use among adolescents young adults and older adults in Chiang Mai, Thailand: findings from a population based survey. BMC Res Notes.

[65] Willard Cates J, Steiner MJ (2002). Dual protection against unintended pregnancy and sexually transmitted infections: what is the best contraceptive approach?. Sex Transm Dis.

[66] Khalesi ZB, Simbar M, Azin SA (2017). A qualitative study of sexual health education among Iranian engaged couples. Afr Health Sci.

[67] Alimoradi Z, Kariman N, Simbar M, Ahmadi F (2017). Empowerment of adolescent girls for sexual and reproductive health care: a qualitative study. Afr J Reprod Health.

[68] Weinstein RB, Walsh JL, Ward LM (2008). Testing a new measure of sexual health knowledge and its connections to students' sex education, communication, confidence, and condom use. Int J Sex Health.

[69] Chu CM, Arya LA, Andy UU (2015). Impact of urinary incontinence on female sexual health in women during midlife. Women’s Midlife Health.

[70] Naderyanfar F, Kadkhodaei F, Mansouri A, Rezaei Keykhahi K, Nehbandani S (2019). Evaluation of distance education using educational videos on the sexual function of women with diabetes type II. J Diabetes Nurs.

[71] Mansouriyeh N, Poursharifi H, Taban SM, Seirafi M (2018). The relationship between socioeconomic status and self-care in patients with heart failure: the role of illness related worries mediator. Q J Nurse Phys War.

[72] Saberi N, Akbari SAA, Mahmoodi Z, Nasiri M (2018). Model for the relationship between sexual function and social determinants of health: path analysis. Koomesh..

[73] Lou C-H, Wang B, Shen Y, Gao E-S (2004). Effects of a community-based sex education and reproductive health service program on contraceptive use of unmarried youths in Shanghai. J Adolesc Health.

[74] Canning D, Schultz TP (2012). The economic consequences of reproductive health and family planning. Lancet.

[75] Price NL, Hawkins K (2007). A conceptual framework for the social analysis of reproductive health. J Health Popul Nutr.

[76] Rabieepur S, Ebrahimi M, Sadeghi E (2015). Relationship between sexual health and contraception methods in women. J Mazandaran Univ Med Sci.

[77] Bahrami N, Soleimani M, Shraifnia H, Masoodi R, Shaigan H. Female sexual satisfaction with different contraceptive methods. Iran J Nurs (2008-5923). 2012;25(76).

[78] McLaurin-Jones TL, Lashley M-B, Marshall VJ (2017). Using qualitative methods to understand perceptions of risk and condom use in African American college women: implications for sexual health promotion. Health Educ Behav.

[79] Parsapure R, Rahimiforushani A, Majlessi F, Montazeri A, Sadeghi R, Garmarudi G (2016). Impact of health-promoting educational intervention on lifestyle (nutrition behaviors, physical activity and mental health) related to vaginal health among reproductive-aged women with vaginitis. Iran Red Crescent Med J.

[80] Ebrahimi TM, Ghofranipour F, Hajizadeh E, Abedini M (2015). Assessment of educational needs among women of reproductive age with common genital tract infections (vaginitis): the first step for developing a self-care educational package. Int J Women’s Health Reprod Sc.

[81] Sanjari S, Mohammadi SM (2017). The impact of education on the prevention of vaginal infection based on the PRECEDE-PROCEED model. J Rafsanjan Univ Med Sc.

[82] Wan Muda WM, Wong LP, Tay ST (2018). Prevention practices of vaginitis among Malaysian women and its associated factors. J Obstet Gynaecol.

